# Modeling Spitz melanoma in zebrafish using sequential mutagenesis

**DOI:** 10.1242/dmm.049452

**Published:** 2022-08-26

**Authors:** Jeffrey K. Mito, Margaret C. Weber, Alexandra Corbin, George F. Murphy, Leonard I. Zon

**Affiliations:** 1 Stem Cell Program and Division of Hematology/Oncology, Boston Children's Hospital, Boston, MA 02215, USA; 2 Department of Pathology, Brigham and Women's Hospital, Boston, MA 02215, USA; 3 Department of Stem Cell and Regenerative Biology, Harvard University, Cambridge, MA 02138, USA; 4 Howard Hughes Medical Institute, Harvard Medical School, Boston, MA 02215, USA

**Keywords:** Zebrafish, Cancer, Melanoma, Electroporation, Spitz neoplasm

## Abstract

Spitz neoplasms are a diverse group of molecularly and histologically defined melanocytic tumors with varying biologic potentials. The precise classification of Spitz neoplasms can be challenging. Recent studies have revealed recurrent fusions involving multiple kinases in a large proportion of Spitz tumors. In this study, we generated a transgenic zebrafish model of Spitz melanoma using a previously identified *ZCCHC8-ROS1* fusion gene. Animals developed grossly apparent melanocytic proliferations as early as 3 weeks of age and overt melanoma as early as 5 weeks. By 7 weeks, *ZCCHC8-ROS1* induced a histologic spectrum of neoplasms ranging from hyperpigmented patches to melanoma. Given the swift onset of these tumors during development, we extended this approach into adult fish using a recently described electroporation technique. Tissue-specific expression of *ZCCHC8-ROS1* in adults led to melanocyte expansion without overt progression to melanoma. Subsequent electroporation with tissue-specific CRISPR, targeting only *tp53* was sufficient to induce transformation to melanoma. Our model exhibits the use of sequential mutagenesis in the adult zebrafish, and demonstrates that *ZCCHC8-ROS1* induces a spectrum of melanocytic lesions that closely mimics human Spitz neoplasms.

## Abstract (precis)

**Summary:** We describe the first animal model of Spitz neoplasms and demonstrate its use for modeling sequential mutagenesis and its potential for studying melanocyte development *in vivo*.

## INTRODUCTION

Spitz neoplasms are an uncommon group of difficult-to-classify melanocytic tumors with both benign and malignant clinical outcomes. The majority of Spitz neoplasms are benign and termed Spitz nevi. By contrast, clinically aggressive tumors with marked cytologic atypia have been variably referred to as Spitz melanoma or malignant Spitz tumors. However, the distinction between a benign Spitz nevus and a Spitz melanoma is not always possible on morphologic grounds alone, which has given rise to the terms ‘atypical Spitz tumor’ or ‘Spitz tumor of unknown malignant potential’ (Barnhill et al., 1999; Reed et al., 1975; Smith et al., 1989).

Recent advances in our molecular understanding of Spitz neoplasms have identified recurrent fusions that involve, several kinases, such as *ALK*, *ROS1* and *MAP3K8*, among others (Raghavan et al., 2020; Wiesner et al., 2014). These observations have led several groups to suggest that integrating next-generation sequencing (NGS) and histologic analysis of Spitz neoplasms will provide improved risk stratification for patients (Quan et al., 2020; Zarabi et al., 2020). Despite a number of studies attempting to link clinical outcomes of Spitz neoplasms to NGS results, these tumors are uncommon, making it challenging to determine the importance of specific genetic alterations for the biologic potential of these tumors.

*ROS1* fusions account for ∼10-30% of kinase fusions in Spitz neoplasms and have been identified in benign Spitz nevi, atypical Spitz tumors and Spitz melanoma (Cesinaro et al., 2021; Gerami et al., 2020; Wiesner et al., 2014). *ROS1* encodes a receptor tyrosine kinase, the function of which in humans is not well defined (Drilon et al., 2021). *ROS1* fusion genes are best characterized in the context of lung adenocarcinoma, where they occur in 1-2% of patients (Gainor and Shaw, 2013). Numerous *ROS1* fusion partners have been identified. All of these fusions result in retention of the C-terminal kinase domain of *ROS1* fused to one of several N-terminal partners that are thought to provide a dimerization function, resulting in ROS1 activation and downstream signaling through the MAPK, PI3K and JAK/STAT pathways, among others (Davies and Doebele, 2013).

This study aims to characterize the role of the recurrent *ZCCHC8-ROS1* fusion gene in the development of Spitz neoplasms. Given that Spitz neoplasms occur in patients of all ages, we explore the oncogenic activity of this fusion protein in development and adult zebrafish. We show that, in developing fish, expression of *ZCCHC8-ROS1* alone leads to an atypical melanocytic proliferation and eventual melanoma formation that is irrespective of tumor suppressor loss. By contrast, expression of *ZCCHC8-ROS1* in adult melanocytes results in the rapid formation of pigmented patches, without overt progression to melanoma. We expand upon the later observation through sequential mutagenesis of *tp53* in adult fish in order to show that subsequent tumor suppressor loss results in aggressive melanoma formation.

## RESULTS

### *ZCCHC8-ROS1* expression leads to aberrant melanocyte development and patterning

**Fig. 1.**
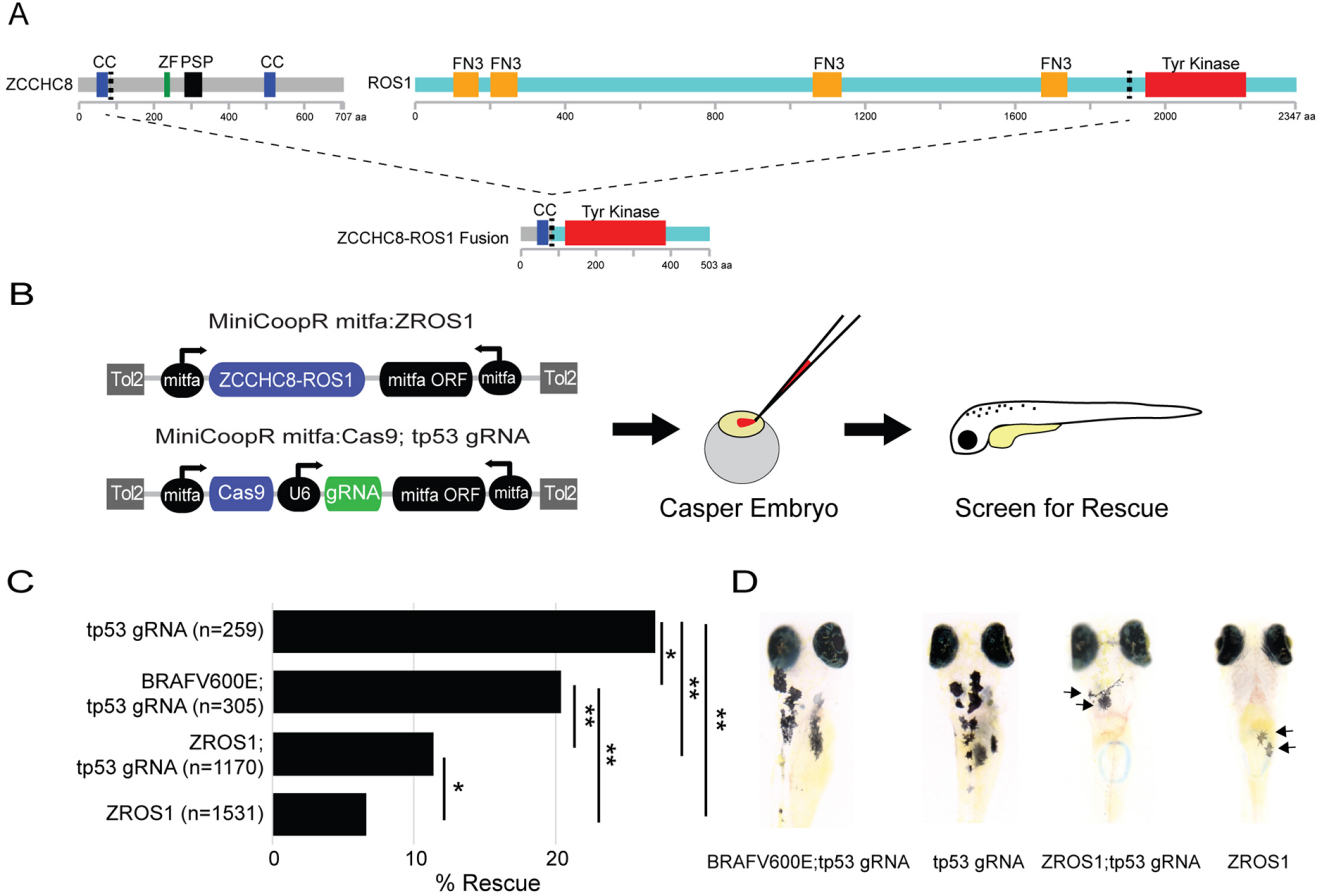
**Expression of the ZCCHC8-ROS1 fusion protein in zebrafish impairs melanocyte rescue.** (A) Diagram of the ZCCHC8-ROS1 fusion protein, which consists of the first coiled-coil domain of ZCCHC8 and the C-terminal tyrosine kinase domain of ROS1. CC, ZCCHC8 coiled-coil region (blue); ZF, zinc finger (green); PSP, Proline rich-domain (black); FN3, Fibronectin type-III (orange); Tyr Kinase, ROS1 tyrosine kinase domain (red). (B) Schematic of the overexpression strategy into *casper* zebrafish embryos using MiniCoopR (MCR):ZROS1. Top: MCR:ZROS1. Bottom: MCR:Cas9;gRNA tp53. (C) Rescue efficiency of various *MCR* vectors injected into *casper* zebrafish embryos, determined by the percentage of embryos showing at least one melanocyte (*P<0.001, **P<0.0001, Chi-square test using aggregate data from multiple experiments, *n*=3). (D) Representative images of *casper* zebrafish embryos showing rescue 5 days post fertilization (arrows denote rare melanocytes).

The *ZCCHC8-ROS1* fusion gene (ZROS1) has previously been identified in patient samples (Cesinaro et al., 2021; Wiesner et al., 2014). It consists of the first coiled-coil domain within the N-terminal fragment of ZCCHC8, a component of the nuclear exosome targeting complex, joined to the kinase domain of ROS1 (Fig. 1A). To test the functional role of ZROS1 in melanocyte development and melanoma progression *in vivo*, we introduced it into the transposon-based vector MiniCoopR (hereafter referred to as MCR) (Ceol et al., 2011), yielding ZROS1-expressing vector MCR:ZROS1. We used *casper* zebrafish, which lack melanocytes and iridophores (White et al., 2008). In this system, melanocytes do not develop, owing to a germline mutation in the *mitfa* gene. Introduction of the MCR:ZROS1 vector reintroduces *mitfa* as well as the ZROS1 fusion gene, which leads to melanocyte rescue and expression of ZROS1 protein in a tissue-specific manner via the *mitfa* promoter (Fig. 1B). Fish injected with either MCR:ZROS1, MCR:BRAF^V600E^, which overexpresses human BRAF^V600E^ via the *mitfa* promoter, or MCR:Cas9;gRNA tp53, which inactivates *tp53* in a tissue-specific manner, were screened for melanocyte rescue at day 4-5 and demonstrated a greatly reduced rescue efficiency in embryos injected with MCR:ZROS1 – irrespective of co-injection with MCR:Cas9;gRNA tp53 – compared to those that received MCR:Cas9;gRNA tp53 alone (*P*<0.001, Fig. 1C). This was similarly reflected in the reduced number of melanocytes identified when animals were screened for melanocyte rescue (day 4-5, Fig. 1D), suggesting that melanocyte development is greatly hindered by expression of ZROS1 during embryogenesis.

**Fig. 2.**
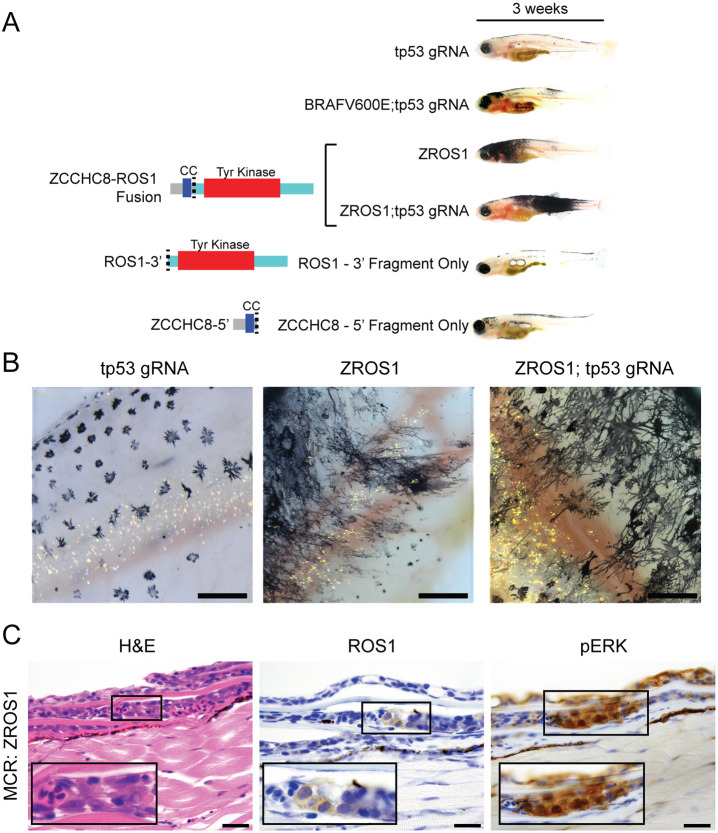
**ZCCHC8-ROS1 leads to hyperpigmented patches and atypical melanocytic proliferations.** (A) Expression of ZCCHC8-ROS1 protein in *casper zebrafish* leads to large hyperpigmented patches by 3 weeks in contrast to control vectors (MCR:BRAF^V600E^, MCR:Cas9;gRNA tp53, MCR:ZCCHC8-5′, expressing just the 5′ portion of the ZROS1 fusion gene derived from ZCCHC8, and MCR:ROS1-3′, expressing just the 3′ portion of the ZROS1 fusion gene derived from ROS1). (B) Microscopic images of 3-week-old *casper* zebrafish, showing melanocyte morphology. The edges of these patches, which tended to form on the dorsal body or head, show melanocytes with hyper-dendritic patterns compared to those in *tp53*-deficient melanocytes. (C) Microscopic examination of the hyperpigmented areas shown in B. Boxed areas show atypical melanocytic proliferations (shown magnified in each inset) and immunostaining for human ROS1 and phosphorylated ERK (pERK), the latter indicating activation of MAPK signaling. Scale bars: 200 μm (B), 20 μm (C).

Despite the relative initial paucity of embryonic melanocytes, ZROS1-injected fish developed large pigmented patches by three weeks (Fig. 2A). This was not seen in controls injected with MCR vectors containing either only the 5′ fragment of ZCCHC8 or the 3′ fragment of ROS1. Upon examination of melanocytes 3 weeks post injection, we observed a highly dendritic morphology that was absent in melanocytes lacking only *tp53* (Fig. 2B). These pigmented patches tended to initially form along the dorsal body surface and head. Histologic examination of these fish at 5-7 weeks demonstrated a microscopically apparent atypical melanocytic proliferation underlying the scales, which was confirmed by detection of human ROS1 and downstream activation of the MAPK pathway, as shown by immunostaining for phosphorylated ERK (phospho-ERK) (Fig. 2C). These results suggest that ZROS1 is responsible for the rapid expansion of melanocytes, despite the relative initial paucity early in development.

### *ZCCHC8-ROS1* induces rapid tumor formation

**Fig. 3.**
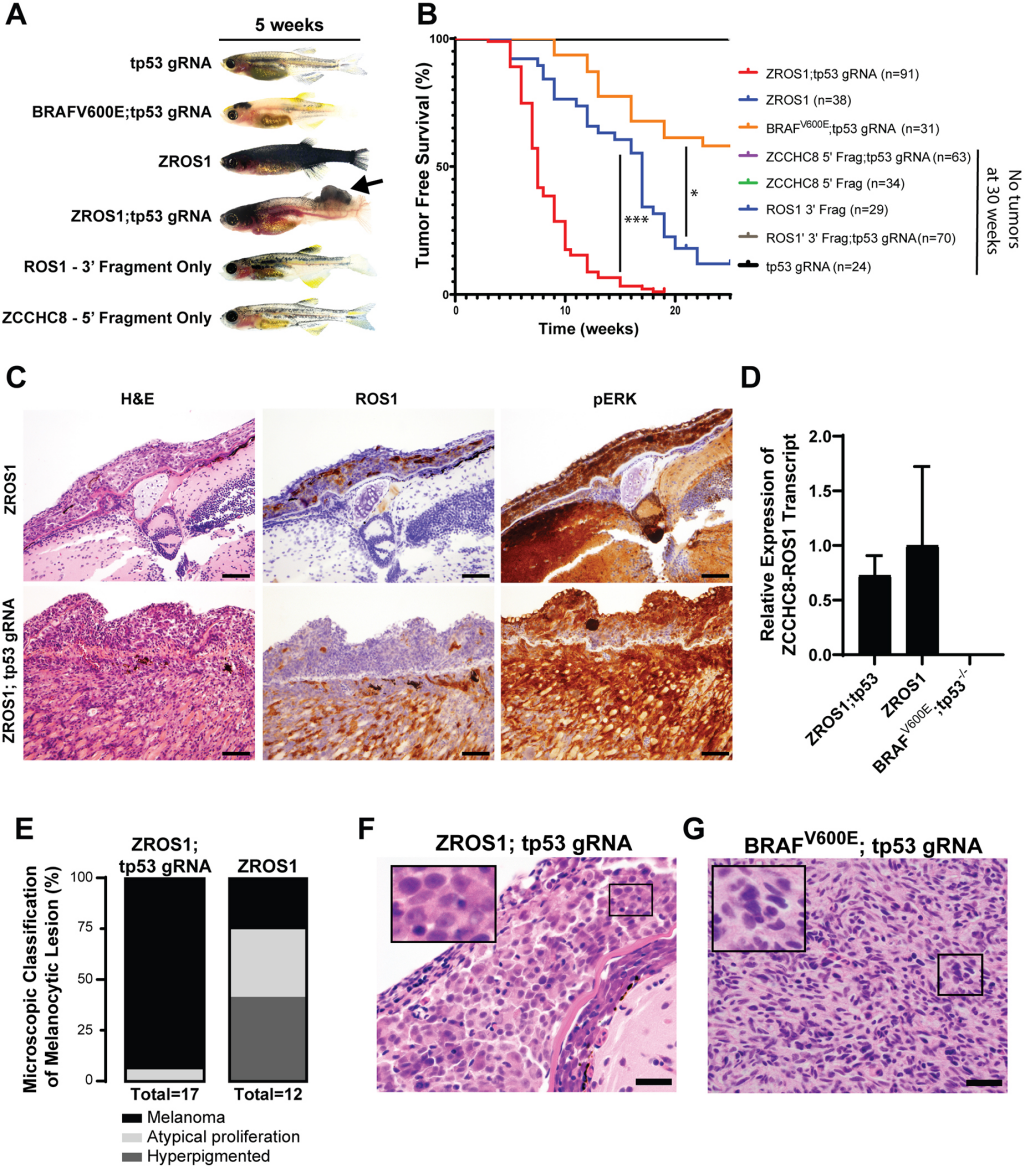
**ZCCHC8-ROS1 induces a spectrum of melanocytic lesions.** (A) Representative images of *casper* zebrafish injected at 5 weeks with different MCR vectors as indicated. The arrow indicates a grossly apparent tumor. (B) Tumor-free survival (up to 25 weeks) of fish injected with different MCR vectors as indicated, plotted using the Kaplan–Meier estimator. Fish injected with MCR:ZROS1;MCR:Cas9;gRNA tp53 (red) and MCR:ZROS1 (blue) show rapid tumor acceleration compared to animals injected with MCR:BRAF^V600E^;MCR:Cas9;gRNA tp53 (orange) (*P<0.001, ***P<0.0001, log-rank test). (C) Representative histologic and immunohistochemical images of 7-weeks-old zebrafish, showing a grossly apparent atypical melanocytic proliferation in MCR:ZROS1-injected animals (upper panels), which does not invade the underlying tissue, and a deeply infiltrative melanoma in MCR:ZROS1;MCR:Cas9;gRNA tp53-injected animals (lower panels). (D) Plotted is the relative expression of ZCCHC8-ROS1 transcripts from animals described in C. The ZROS1 fusion transcript was readily detected by qRT-PCR in tumors derived from both ZROS1 (*n*=3) and ZROS1;tp53^−/−^ (*n*=3) (mean±s.d., all samples were run in technical triplicates). (E) Plotted is the classification of a histologically defined melanocytic lesion by genotype at 7 weeks. ZROS1;tp53^−/−^ *casper* zebrafish demonstrated a higher rate of melanomas compared to fish expressing ZROS1 alone, which demonstrated a spectrum of melanocytic lesions ranging from hyperpigmentation to atypical proliferations and a small subset of melanomas. (F,G) Microscopic examination of ZROS1-driven tumors (F), which tended to have a distinctly epithelioid morphology with moderate amounts of cytoplasm, prominent nucleoli and distinct cell borders, contrasting with that of BRAF^V600E^;tp53^−/−^-derived tumors (G), which showed a more varied epithelioid to spindled morphology with non-distinct cell borders. Boxed areas show prominent nucleoli (magnified in insets). Scale bars: 50 μm (C), 20 μm (F,G).

The aberrant pigmentation phenotype of the ZROS1 fish continued throughout development and gross tumors were observed as early as 5 weeks of age – in contrast to 9 weeks for the earliest tumors in BRAF^V600E^;tp53^−/−^ animals (Fig. 3A). Both ZROS1;tp53^−/−^ and ZROS1 fish had rapid tumor onset relative to BRAF^V600E^;tp53^−/−^ zebrafish. In particular, ZROS1;tp53^−/−^ showed complete penetrance of tumor formation by 20 weeks of age, whereas ZROS1 alone led to highly penetrant tumors with delayed kinetics (Fig. 3B, *P*<0.0001). No tumors were apparent in the first 30 weeks of observation for controls containing either the 3′-ROS1 fusion fragment or the 5′-ZCCHC8 fusion fragment. Expression of ZROS1 alone led to grossly apparent atypical melanocyte proliferations akin to atypical Spitz tumors by 5-7 weeks of age (Fig. 3C, upper panel). By contrast, at 7 weeks, ≥50% ZROS1;tp53^−/−^ fish developed grossly apparent melanomas (Fig. 3B), which were highly aggressive and infiltrative (Fig. 3C, lower panel). In either case, despite likely having a small contribution from contaminating genomic DNA, the ZROS1 fusion transcript was readily detectable by qRT-PCR (Fig. 3D). Histologic examination at 7 weeks, found at least superficially invasive melanomas in almost all ZROS1;tp53^−/−^ fish but only a small portion in ZROS1 fish. Based on morphologic analysis, the latter had a mixture of hyperpigmented areas without definite melanocytic expansion, and atypical proliferations leading to grossly apparent lesions and invasive melanomas (Fig. 3E). The melanocytes in the ZROS1;tp53^−/−^ model tended to show a distinctly epithelioid morphology with very distinct nucleoli (Fig. 3F) compared to those in the BRAF^V600E^;tp53^−/−^ model (Ceol et al., 2011), which tended to show a very non-distinct epithelioid to spindled morphology without well-defined cell borders (Fig. 3G). These findings demonstrate that ZROS1 expression leads to rapid onset of a morphologically distinct melanoma from the BRAF^V600E^-driven model.

### Rapid melanoma initiation in adult zebrafish using the TEAZ technique

**Fig. 4.**
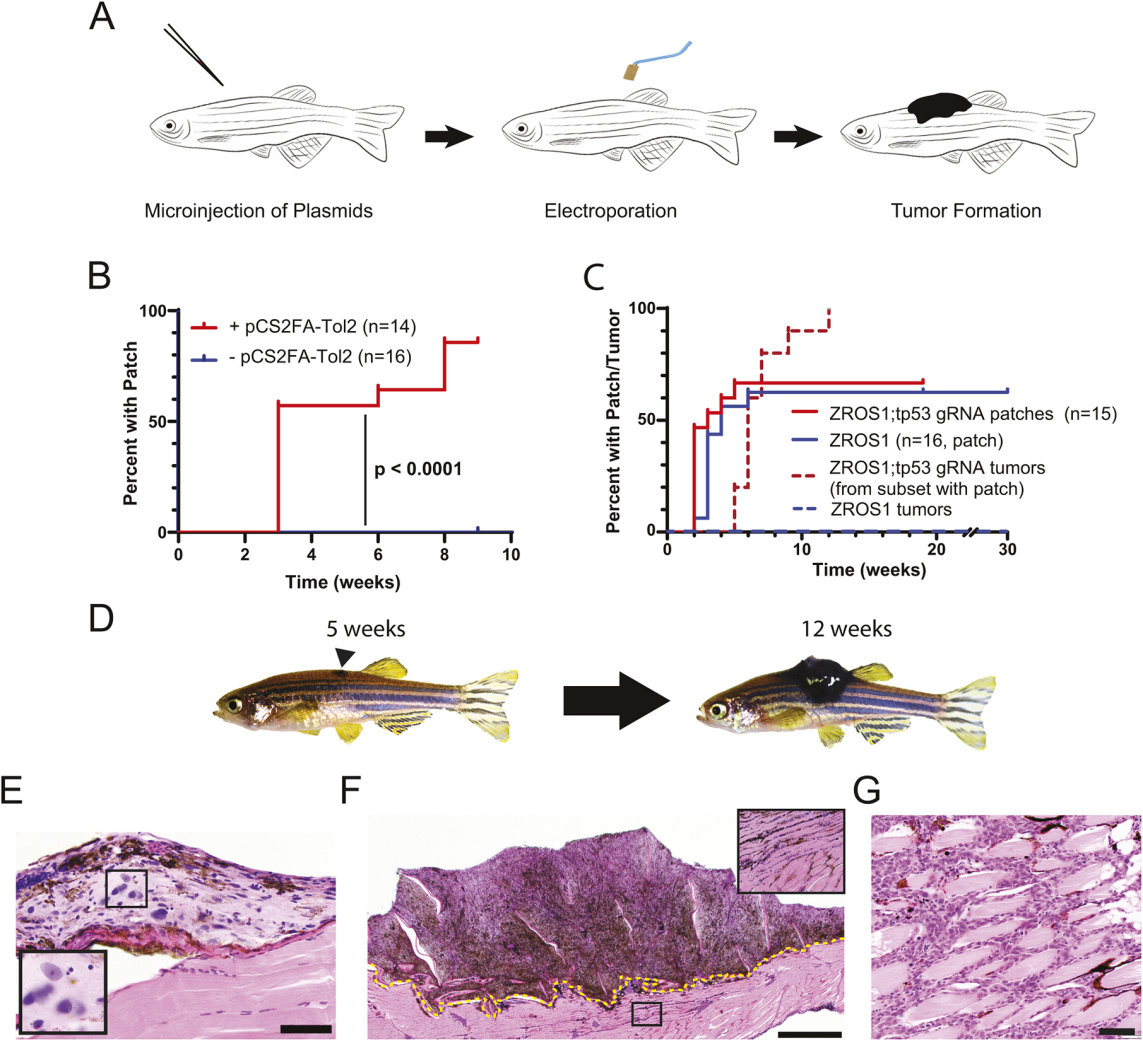
**Electroporation in wild-type adult zebrafish readily generates melanomas.** (A) Schematic of Transgene Electroporation in Adult Zebrafish (TEAZ) process. (B) Addition of pCS2FA-Tol2 to MCR:ZROS1 (red) greatly improves the efficiency of electroporation, as determined by the formation of a pigmented patch (*P*<0.0001, log rank test). (C) Electroporation of MCR:ZROS1 leads to efficient patch formation (red and blue solid lines), whereas addition of MCR:Cas9;gRNA tp53 leads to highly penetrant tumors in all animals that develop pigmented patches (red dashed line). Fish electroporated with MCR:ZROS1 did not develop tumors up to 30 weeks. (D) Representative images of electroporated wild-type TU fish at 5 and 12 weeks, with a tumor clearly visible at 12 weeks. (E-G) Representative histologic images of ZROS1;tp53^−/−^ TEAZ fish. An early, slightly raised lesion with cytologic atypia (boxed area, shown magnified in inset) that lacks invasion in shown in E. Panel F shows a large exophytic tumor with foci of invasive and pigmented cells in the underlying skeletal muscle (boxed area, shown magnified in inset). The dashed line indicates the point of invasion into the underlying skeletal muscle. Invasive tumors (G) frequently show a highly infiltrative pattern into the underlying skeletal muscle. Scale bars: 20 μm (E), 500 μm (F), 50 μm (G).

Recently, Callahan and colleagues have described Transgene Electroporation in Adult Zebrafish (TEAZ), a technique to generate melanomas within adult zebrafish in a temporally and spatially restricted manner (Callahan et al., 2018). The initial report of TEAZ used multiple transgenes to develop robust tumors and, ultimately, required protein kinase BRAF^V600E^ , i.e. BRAF in which valine (V) at position 600 had been mutated to glutamic acid (E), in addition to loss of *rb1* in a *tp53*-deficient background. We adapted the TEAZ technique for use in wild-type *Tübingen* (TU) zebrafish by taking advantage of the MCR system, the vectors of which contain *Tol2* sequences (Fig. 4A). We hypothesized that we could improve the efficiency of TEAZ by leveraging these *Tol2* sites by expressing *Tol2* via a ubiquitous promoter introduced during the electroporation procedure. As anticipated, the addition of a vector driving expression of *Tol2* (i.e. pCS2FA-Tol2) to the electroporation mixture containing MCR:ZROS1 dramatically improved the TEAZ process in wild-type TU zebrafish (Fig. 4B, *P*<0.0001). The presence of pre-existing mature adult melanocytes was essential to the high efficiency of the TEAZ process because *casper* fish showed significantly reduced patch formation compared with that of wild-type TU fish (Table 1, *P*<0.0001, Chi-square test). As such, we used wild-type TU fish for all subsequent experiments. ZROS1 alone readily induced pigmented patches at the site of electroporation in adult zebrafish, which was not seen when electroporating MCR:BRAF^V600E^ (Table 1). In contrast to ZROS1 expression during zebrafish development, these patches did not readily progress to melanoma. However, electroporation of MCR:ZROS1 together with MCR:Cas9;gRNA tp53 to inactivate *tp53*, led to all fish with patches going on to develop tumors within 12 weeks (Fig. 4C,D). Histologically, these tumors recapitulate features of a progressive lesion with early patches showing a highly atypical proliferation of melanocytes that do not invade into the underlying skeletal muscle (Fig. 4E). As the lesions progress into large exophytic tumors (Fig. 4F), they infiltrate the underlying skeletal muscle (Fig. 4F,G). These results suggest that two genetic events are sufficient to initiate melanoma in wild-type TU zebrafish.

**Table 1.**
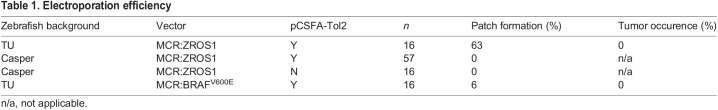
Electroporation efficiency

**Fig. 5.**
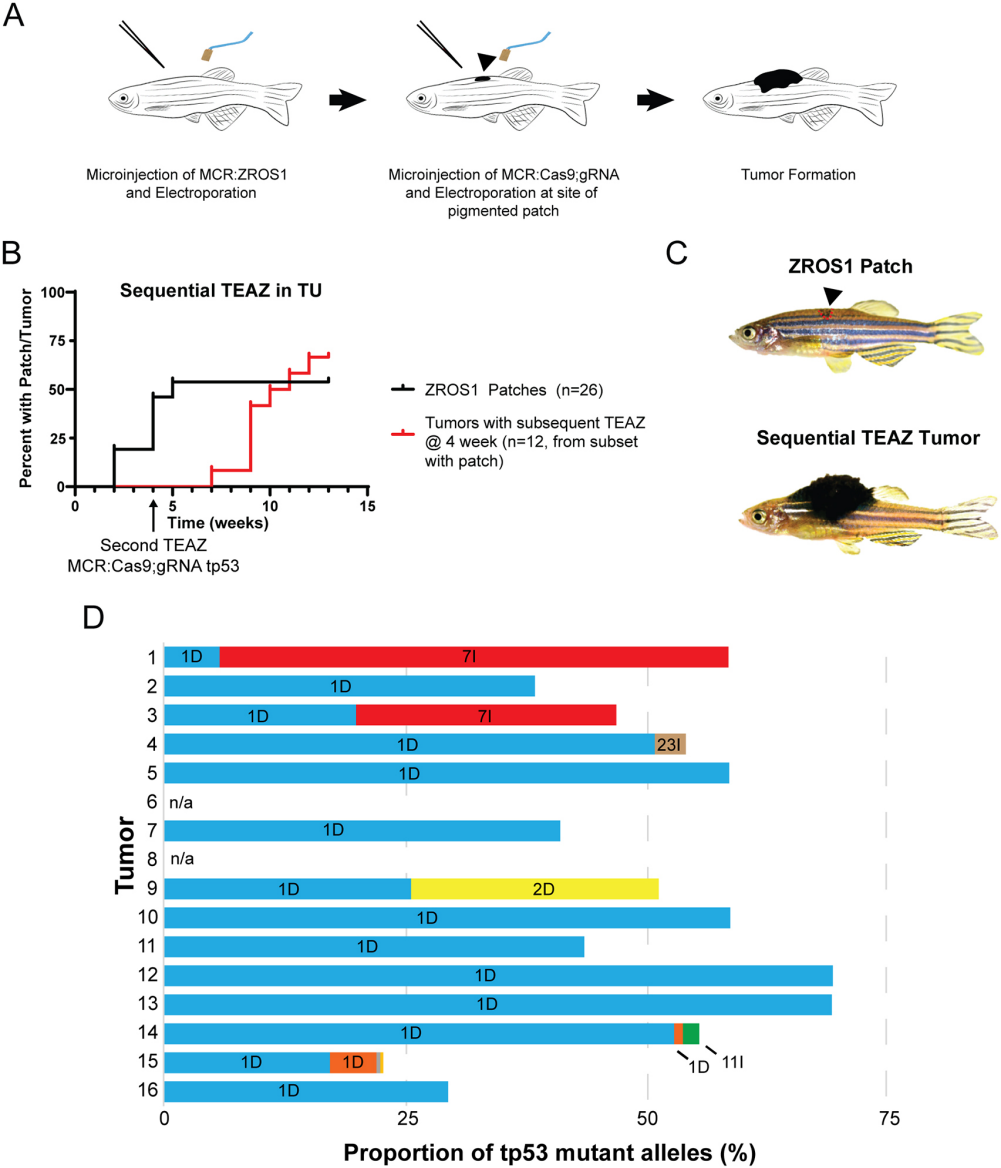
**Sequential mutagenesis reliably induces clonal tumors.** (A) Schematic of the sequential mutagenesis experiment in TU zebrafish. The arrowhead indicates the formation of a pigmented patch. (B) Electroporation of MCR:Cas9;gRNA tp53 into pigmented patches (black line) 4 weeks after the initial TEAZ. The red line denotes the proportion of fish with tumors generated from all fish with patches that underwent the second electroporation with MCR:Cas9;gRNA tp53. (C) Representative images of fish that had undergone sequential TEAZ (arrowhead and red dashed line denotes a pigmented patch). (D) Targeted sequencing of the *tp53* CRISPR site. Most tumors show a single and dominant clonal edit (colors represent a specific mutant allele, 1D, deletion of one pair; 2D, deletion of two pairs; 7I, insertion of seven base pairs; 11I, insertion of eleven base pairs; 23I=insertion of twenty-three base pairs; n/a, not applicable (wild type sequence only).

Given the lack of overt tumor formation in adult fish electroporated with MCR:ZROS1 alone, and the apparent progressive nature of the ZROS1;tp53^−/−^ TEAZ fish, we hypothesized that this model represents a robust system for sequential mutagenesis in the zebrafish (Fig. 5A). To this end, we first electroporated adult TU fish with MCR:ZROS1 and waited for pigmented patches to form (at ∼4 weeks post electroporation). Fish with patches were subsequently electroporated again with MCR:Cas9;gRNA tp53, resulting in tumor formation in the majority of fish that had developed pigmented patches upon electroporation of MCR:ZROS1 alone (Fig. 5B (red line), Fig. 5C). Sequencing of the *tp53* CRISPR site in fish that developed tumors after sequential TEAZ demonstrated that the majority of tumors comprised single dominant CRISPR edit (12 of 16) or similar variant allele fractions of two edits, suggesting a single clone (two of 16) (Fig. 5D). Two additional tumors had no detectable variant alleles at the *tp53* CRISPR site. These data suggested that these tumors typically arise from a single dominant clone.

## DISCUSSION

Investigating Spitz neoplasms has been challenging, owing to the rarity of these tumors and lack of cell lines or animal models. In this study, we describe the first animal model of Spitz tumors. *ZCCHC8-ROS1* (ZROS1) fusion gene expressed under the *mitfa* promoter is sufficient to drive these tumors. One of the limitations we initially encountered with this model was with embryo injections of the ZROS1 transgene limiting melanocyte rescue (∼7%, Fig. 1C). This made these injections an incredibly labor-intensive enterprise that often yielded only a handful of fish with melanocyte rescue. Previous *in vitro* work has suggested a role for JAK/STAT signaling, which is a known downstream target of ROS1 (Crescenzo et al., 2015), in inhibiting melanogenesis (Choi et al., 2013). This might explain the poor melanocyte rescue initially seen in ZROS1-injected embryos (Fig. 1C,D). Although initially reminiscent of zebrafish with inactivating *kita* mutations, which have significant reductions in early melanocytes (Hultman et al., 2007), this phenomenon was transient; large hyperpigmented patches developed on the majority of MCR:ZROS1-injected zebrafish that showed any degree of melanocyte rescue by 3 weeks of age (Fig. 2A). This suggested that these heavily pigmented patches were derived not from the initial wave of embryonic melanocytes but, rather, a subsequent wave of melanocytes probably derived from postembryonic melanophore progenitors (Dooley et al., 2013). Interestingly, we noticed a hyper-dendritic appearance to the melanocytes at the edge of these pigmented patches. One possible explanation for this morphology is the activation of downstream Rho signaling, a known downstream target of ROS1 fusion proteins and a previously described regulator of melanocyte dendricity (Kim et al., 2010; Zeng et al., 2000). These observations suggest that the dysregulation of ROS1-targeted pathways in melanocyte development can have broader implications for pigmentation, developmental patterning and melanocyte biology, which would be amenable to further study in this model system.

Over time, ZROS1-injected zebrafish almost universally develop large invasive tumors, regardless of loss of *tp53* (Fig. 3B). At 7 weeks of age, ZROS1 fish had a spectrum of morphologic changes ranging from overt melanoma to hyperpigmented patches (Fig. 3E). This diversity of morphologies in the MCR:ZROS1 fish closely overlaps with the spectrum of Spitz neoplasms seen in patients (Menezes and Mooi, 2017), including a tendency towards a striking epithelioid morphology (Fig. 3F) (Cesinaro et al., 2021; Gerami et al., 2020). This is in contrast to the BRAF^V600E^;tp53^−/−^ model, in which melanomas tend to have more-variable histologic appearance and, when present, a less distinct epithelioid cytomorphology (Fig. 3G) (Ceol et al., 2011). Additionally, co-injection of MCR:ZROS1 and MCR:Cas9;gRNA tp53 led to rapid tumor generation with aggressive melanomas in as few as 5 weeks (Fig. 3A,B), demonstrating that the loss of a tumor suppressor accelerates tumorigenesis. This is in keeping with *TP53* mutations in humans being progressively more common – from Spitz nevi, to atypical Spitz tumors and Spitz melanomas (Dal Pozzo and Cappellesso, 2022).

Although Spitz tumors are more frequent in adolescents, they occur in patients of all ages. Given the rapid development of tumors when injected into the one-cell embryo, we sought to study the ability of ZROS1 to induce tumors in adult zebrafish – in which the melanocyte pool is derived from differentiated adult melanocytes – by applying the recently described TEAZ technique (Callahan et al., 2018). Our data demonstrate that the efficiency of TEAZ can be greatly improved by co-electroporation with a vector expressing *Tol2*; the simple injection and electroporation process is easily learned and requires limited specialized equipment beyond the electroporator. This addition is likely to drive integration of the various MCR vectors – which contain Tol2 sites – into the zebrafish genome, leading to a greater than 10-fold enhancement of the TEAZ process efficiency in wild-type zebrafish (Fig. 4A). Interestingly, in contrast to the work by Callahan and colleagues, we did not observe an acceleration in tumor development when converting from embryo injections to TEAZ. This could be a consequence of using TU fish with pre-existing melanocytes or, perhaps, a function of the ZROS1 fusion gene itself.

Electroporation of MCR:ZROS1 alone into adult zebrafish induced variably sized hyperpigmented patches, the majority of which formed within 5 weeks of electroporation (Fig. 4C). Pigmented patches were rarely seen when electroporating MCR:BRAF^V600E^ alone (Table 1). This raises the possibility that the pigmented patches seen with TEAZ of MCR:ZROS1 result from a phenotypic change in pre-existing adult melanocytes and progenitors, leading to an increased pigmentation and disrupted patterning without significant proliferation – as TEAZ of MCR:ZROS1 alone did not induce formation of grossly raised tumors, even when following fish out beyond 20 weeks. By contrast, co-electroporation with MCR:Cas9;gRNA tp53 led to robust and rapid formation of grossly apparent tumors in every animal that formed a pigmented patch (Fig. 4C). These data strongly suggest that cooperating loss of a tumor suppressor is needed to give rise to Spitz melanomas. Previous sequencing studies of human Spitz melanomas have made similar observations, i.e. that inactivation of tumor suppressors, such as *CDKN2A*, frequently occur in Spitz melanomas (Raghavan et al., 2020). The role of *TP53* in the development of human Spitz melanomas is less clear. In one study of 40 RAF1-fusion melanomas, 13% (five out of 40) showed inactivating *TP53* mutations. In this particular study, a 60% loss of *CDKN2A* (24 out of 40) and/or 62% with activating *TERT* promoter mutations (23 out of 37) were more common genetic events (Williams et al., 2020). Another study that sequenced nine Spitz melanomas with non-ROS1 rearranged driver mutations did not identify any *TP53* mutations (Raghavan et al., 2020). Moreover, loss of tumor suppressor can also be seen in benign Spitz tumors (Quan et al., 2020), suggesting that other somatic alterations, such as the more-common mutations of the *TERT* promoter or the overall mutational burden, may be better markers of clinical outcomes for these patients (Lee et al., 2015).

We also believe that our work defines the minimum number of genetic elements that are needed to drive melanoma initiation in wild-type adult fish: a single oncogene and the loss of a tumor suppressor. However, we do not believe that this applies to every combination of oncogene and tumor suppressor – as previously demonstrated by Callahan and colleagues – or that it is likely to occur in zebrafish from other genetic backgrounds (Callahan et al., 2018). Additionally, we have attempted to substitute other tumor suppressors, such as *cdkn2a*, for *tp53* in the second TEAZ step and found significantly lower penetrance. This suggests that specific oncogene and tumor suppressor combinations are able to necessitate additional ‘hits’ to initiate melanoma formation. This is evident from injections of MCR:ZROS1 into *casper* zebrafish embryos, which led to melanocytic lesions with a striking epithelioid morphology irrespective of targeting *tp53* (Fig. 3). By contrast, TEAZ-generated tumors in TU zebrafish required additional targeting of *tp53*, and the resulting tumors tended to show greater pleomorphism (Fig. 4E,G). It is possible that this greater atypia is related to non-specific genomic alterations induced by the TEAZ and TOL2 insertional process. The identification of a small subset of tumors with sequential TEAZ showing no *tp53* alterations suggests that *tp53* loss is sufficient but not necessary for melanoma progression, raising the possibility of non-specific genetic events that result from the TEAZ/TOL2 process (Fig. 5D). By contrast, no such second hit appears to be necessary in fish injected at the one-cell stage (Fig. 3B). This may be a function of expression in ZROS1 throughout development, which includes a number of embryonic and post-embryonic progenitors that may be more susceptible to transformation than differentiated adult melanocytes and progenitors, for which loss of a tumor suppressor is likely to be necessary in order to develop melanoma.

Although, generally, a clinically indolent disease compared to conventional melanoma (Bartenstein et al., 2019; Quan et al., 2020), Spitz neoplasms can be challenging to correctly diagnose. In addition, atypical Spitz tumors and melanomas occasionally give rise to metastatic tumors (Pol-Rodriquez et al., 2007; Tlougan et al., 2013). Several recent studies have suggested a dual role for both morphologic evaluation as well as next-generation sequencing to help appropriately classify these lesions (Quan et al., 2020; Zarabi et al., 2020). Beyond being the first animal model of Spitz tumors, our findings expand the utility of the TEAZ system for modeling melanoma, by providing a useful adjunct to clinical sequencing data that explores the role of specific mutations in the progression of Spitz neoplasms in either juvenile or adult zebrafish. This system is interchangeable and has the potential to model other fusion gene-driven melanomas, and to act as a test platform for targeted therapies; moreover, it is a model for performing sequential mutagenesis in a spatially and/or temporally restricted fashion.

## MATERIALS AND METHODS

### Zebrafish modeling

All animal work was performed according to Boston Children's Hospital Institutional Animal Care and Use Committee (protocol #20-10-4253R). Tumors were generated as outlined in Ablain et al. (2018). The MiniCoopR construct (MCR) was used to overexpress the *ZCCHC8-ROS1* fusion gene using HiFi assembly (New England Biolabs, Ipswich, MA, USA) with the previously identified *ZCCHC8-ROS1* open reading frame (Wiesner et al., 2014), which was synthesized as a gBlocks gene fragment, i.e. double-stranded 125- to 3000-bp-long DNA fragments, by Integrated DNA technologies (Coralville, IA, USA). The following primers were used to amplify the construct: 5′-TTGGGTACCGGGCCCCCCCTCGAGGATGGCCGCAGAGGTG-3′ and 5′-GTGGATCCCCCGGGCTGCAGGAATTTTAATCAGACCCATC-3′, for assembly into the MCR vector that has previously been assembled using gateway multiple site cloning (Invitrogen, Waltham, MA, USA) with the *mitfa*-promoter, a multiple cloning site and a poly-adenylation site. The vector was linearized using SalI and EcoRI prior to gateway assembly. Briefly, embryo injections were performed using: *MCR:ZCCHC8-ROS1* (25 pg), *MCR:BRAF ^V600E^ , MCR:Cas9; gRNA tp53* or *MCR:ZCCHC8-ROS1* and *MCR:Cas9; gRNA tp53* (12.5 pg each) together with Tol2 transposase mRNA (25 pg) into one-cell *casper* zebrafish embryos. Embryos were scored for melanocytes rescue at 4-5 days post fertilization and rescue compared by Chi-square test. Zebrafish were examined every 1-2 weeks after injection and followed for the development of melanocytic lesions. Tumor-free survival curves were scored once grossly apparent tumors, defined as a pigmented raised lesion above the body plane, had been identified. Kaplan-Meier curves were generated using GraphPad Prism (GraphPad Software, San Diego, CA) and groups compared by log-rank test. For imaging, zebrafish were anesthetized with 4% MS-222 (Western Chemical Incorporated, Ferndale, WA, USA) and images were taken with a Nikon SMZ18 Stereomicroscope for zebrafish ≤5 weeks of age and a Nikon D3100 camera with an AF-S Micro NIKKOR 60 mm lens for older fish (Nikon Corporation, Tokyo, Japan).

### Electroporation

Zebrafish were anesthetized with 4% MS-222, placed in an agarose mold, and 1 µl of the plasmid of interest resuspended in nuclease-free water (500 ng/µl final concentration) was injected into the dorsal subscale space of the zebrafish. When used, pCS2FA-Tol2 represented 10% of the total DNA content (Kwan et al., 2007). Immediately following injection, electrodes were placed on either side of the injection site and electroporation was performed using the ECM 830 Square wave electroporation system (BTX, Holliston, MA, USA) as previously described (Callahan et al., 2018). After injection, fish were transferred to fresh water and allowed to recover. Zebrafish were followed weekly for the development of pigmented patches and raised lesions. Kaplan-Meier curves for pigmented patch- and tumor-free survival curves were generated using GraphPad Prism and compared by log-rank test. For sequential TEAZ, this process was repeated with injection and electroporation of the pigmented patch.

### Histology and immunohistochemistry

Zebrafish were euthanized and fixed in 10% formalin, processed, paraffin-embedded and sectioned (5 µM) after brief surface decalcification. Using standard techniques, Hematoxylin and Eosin staining was performed by the Dana-Farber/Harvard Cancer Center Specialized Histopathology core. Photomicrographs were acquired on an Olympus BX43 microscope with a DP27 camera (Olympus Corporation, Tokyo, Japan). Histologic sections reviewed by two pathologist (J.K.M. and G.F.M, the latter a dermatopathologist). Melanocytic lesions were classified as (1) hyperpigmented, when no atypical melanocytes were identified; (2) atypical proliferations, when there was mild-to moderate cytologic atypia without evidence of local tissue invasion or; (3) melanomas, when highly infiltrative lesions contained overt cytologic atypia. Immunohistochemistry was performed by the Dana-Farber/Harvard Cancer Center Specialized Histopathology core with 5 µm-thick formalin-fixed, paraffin-embedded tissue sections using the Bond III automated staining platform (Leica Biosystems, Wetzlar, Germany). Antibody against phosphorylated ERK (p44/42 MAPK, clone D13.14.4E, cat #4370, 1:1000 dilution, Cell Signaling Technology, Danvers, MA, USA) was utilized after citrate antigen retrieval. The antibody for ROS1 (clone D4D6, cat #3287, 1:250 dilution, Cell Signaling Technology) was utilized after EDTA antigen retrieval. For both, the Refine Detection Kit (Leica Biosystems) was used.

### qRT-PCR

Tumors were manually isolated from MCR:ZCCHC8-ROS1, MCR:BRAF^V600E^;tp53^−/−^ or MCR:ZCCHC8-ROS1;tp53^−/−^ fish, mechanically homogenized and RNA was isolated using the RNeasey kit (Qiagen, Hilden, Germany) according to the manufacturer's instructions. cDNA was synthesized from 2000 ng of total RNA using the High Capacity cDNA reverse transcription kit (Applied Biosystems, Waltham, MA, USA) per manufacturer's instructions. Real-time quantitative reverse transcription PCR (qRT-PCR) was performed using the C1000 thermocycler (Bio-Rad, Hercules, CA, USA) using iTaq Universal SYBR green supermix (Bio-Rad) with the following primers (Invitrogen): *EF1a*, 5′-GCATACATCAAGAAGATCGGC-3′ and 5′-GCAGCCTTCTGTGCAGACTTTG-3′; ZCCHC8-ROS1, 5′-GCGCCGAGAATCAAGAACTTA-3′ and 5′-ACACTTCTCCAAAGGCTCCA-3′. Relative values were calculated using the ΔΔCT method (normalized to EF1a expression).

### CRISPR sequencing

Tumor tissue was isolated from MCR:ZCCHC8-ROS1;tp53^−/−^ tumors that had been generated in *casper* zebrafish using sequential TEAZ and genomic DNA isolated using the Qiagen DNeasy Blood and Tissue Kit. Genomic DNA was amplified with Phusion high-fidelity DNA polymerase (New England Biolabs) with the following primers (Invitrogen): *tp53*, 5′-CTGTGTTTGCCAGGAGTACTTG-3′ and 5′-TATGTGTGTGTATGCGCTTTTG-3′. PCR products were purified using the QIAquick PCR purification kit (Qiagen). Sequencing was performed at the MGH DNA core and analyzed using CRISPResso2 (https://crispresso.pinellolab.partners.org/) (Clement et al., 2019).
